# 928. Analysis of Burn Patient Claims at Verified Burn Centers versus Non-designated Centers Across U.S. Regions

**DOI:** 10.1093/jbcr/irag033.468

**Published:** 2026-04-06

**Authors:** Patrice Sarrazin, Mark Shearer, Sydnee Jackson, Setareh Khani, Rajiv S Sood

**Affiliations:** Philadelphia College of Osteopathic Medicine, Moultrie, GA; Philadelphia College of Osteopathic Medicine, Moultrie, GA; Philadelphia College of Osteopathic Medicine, Moultrie, GA; Philadelphia College of Osteopathic Medicine, Moultrie, GA; Burn and Reconstructive Centers of America, Augusta, GA

## Abstract

**Introduction:**

In the United States, burn injuries represent a significant health issue that requires specialized care to treat the patient efficiently. The American Burn Association (ABA) provides burn center verification to ensure adherence to efficient standards of care and attention to the quality of care. However, there is an uncertainty whether or not verified burn centers receive a proportionally greater share of burn-related claims volume across U.S. regions. This study examines regional variation in burn claims distribution between verified burn centers and non-designated centers to assess potential disparities in utilization based on burn claims data.

**Methods:**

We analyzed national burn claim data, stratified by burn center verification status (verified vs non-designated) and by U.S. Census region (Northeast, Midwest, South—including Southeast, and West). F-tests were conducted to assess equality of variances between region pairs and Welch’s t-tests, or Student’s t-test were used where appropriate to compare mean claim volumes. Chi-square tests were used to evaluate regional differences in the proportion of claims directed to verified versus non-designated centers.

**Results:**

Across verified centers, only the Northeast–West comparison was significant (*p*=.0255), indicating that verified centers in the Northeast and West receive different mean claim volumes. All other verified and non-designated center comparisons were non-significant (*p*>.05).

However, chi-square analysis revealed highly significant regional disparities in the proportion of claims routed to verified centers (*p*<.001 for all regions). Only 20% of burn claims in the Northeast were directed to verified centers compared to 29% in the West with intermediate utilization in the Midwest (26%) and South (21%).

**Conclusions:**

Although regional differences in mean claims volume were observed, these differences were more statistically evident among non-designated centers. Proportional use of verified centers showed significant variation with the Northeast showing the lowest and the West displaying the highest utilization. Chi-square analysis revealed that verified centers were underutilized in some regions compared to others (e.g., the Northeast vs. West).

**Applicability of Research to Practice:**

These findings propose systemic variation in access to verified burn care and highlight the need to further explore factors such as referral pathways, facility availability, and policy barriers influencing regional access to specialized trauma care.

**Funding for the study:**

N/A.

